# Training Rarámuri Criollo Cattle to Virtual Fencing in a Chaparral Rangeland

**DOI:** 10.3390/ani15152178

**Published:** 2025-07-24

**Authors:** Sara E. Campa Madrid, Andres R. Perea, Micah Funk, Maximiliano J. Spetter, Mehmet Bakir, Jeremy Walker, Rick E. Estell, Brandon Smythe, Sergio Soto-Navarro, Sheri A. Spiegal, Brandon T. Bestelmeyer, Santiago A. Utsumi

**Affiliations:** 1 Animal and Range Sciences, New Mexico State University, Las Cruces, NM 88003, USA; saraecma@nmsu.edu (S.E.C.M.); arperea@nmsu.edu (A.R.P.); funkm@nmsu.edu (M.F.); mspetter@nmsu.edu (M.J.S.); mebakir@nmsu.edu (M.B.); bsmythe@nmsu.edu (B.S.); ssoto@nmsu.edu (S.S.-N.); 2 Corta Madera Ranch, Pine Valley, CA 91962, USA; jeremy@cortamadera.com; 3 USDA-ARS Jornada Experimental Range, Las Cruces, NM 88003, USA; rick.estell@usda.gov (R.E.E.); sheri.spiegal@usda.gov (S.A.S.); brandon.bestelmeyer@usda.gov (B.T.B.)

**Keywords:** animal behavior, animal welfare, animal monitoring, grazing distribution, precision livestock management, smart farming

## Abstract

Virtual fencing (VF) offers a promising alternative to conventional or electrified fences for managing livestock grazing distribution. This study evaluated the behavioral responses of 25 Rarámuri Criollo cows fitted with Nofence^®^ collars in Pine Valley, CA, USA. The VF system was deployed in chaparral rangeland pastures. The study included a 14-day training phase followed by an 18-day testing phase. The collar-recorded variables, including audio warnings and electric pulses, animal movement, and daily typical behavior patterns of cows classified into a High or Low virtual fence response group, were compared using repeated-measure analyses with mixed models. During training, High-response cows (i.e., resistant responders) received more audio warnings and electric pulses, while Low-response cows (i.e., active responders) had fewer audio warnings and electric pulses, explored smaller areas, and exhibited lower mobility. Despite these differences, both groups showed a time-dependent decrease in the pulse-to-warning ratio, indicating increased reliance on audio cues and reduced need for electrical stimulation to achieve similar containment rates. In the testing phase, both groups maintained high containment with minimal reinforcement. The study found that Rarámuri Criollo cows can effectively adapt to virtual fencing technology, achieving over 99% containment rate while displaying typical diurnal patterns for grazing, resting, or traveling behavior. These findings support the technical feasibility of using virtual fencing in chaparral rangelands and underscore the importance of accounting for individual behavioral variability in behavior-based containment systems.

## Simple Summary

Virtual fencing is a promising alternative for managing cattle grazing distribution, but differences in learning and behavior may affect the containment success rates. This study aimed to evaluate the effectiveness of virtual fencing Rarámuri Criollo cattle on rangelands. Cows showed varying learning responses to virtual fencing, with proactive animals engaging with the fence more often than less proactive ones. Despite these differences, cows in the two response groups maintained a high containment rate with minimal audio warnings or pulse reinforcements during later testing phases. The study found that Rarámuri Criollo cattle can effectively adapt to virtual fencing technology, achieving over 99% containment rate while displaying typical diurnal patterns for grazing, resting, or locomotory behavior. Therefore, this research highlights the promising potential and flexibility of virtual fencing for extensive beef production.

## 1. Introduction

Virtual fencing technology offers a promising approach to managing livestock grazing without the use of physical barriers. By employing Global Navigation Satellite System (GNSS) in GNSS-enabled collars, virtual fencing allows for dynamic, adaptive control of cattle movement, promoting sustainable grazing and improved pasture utilization [1]. In contrast to traditional fencing, which is costly, labor-intensive, and static, virtual fencing provides a more flexible alternative for adaptive grazing management, which is particularly valuable in extensive rangeland systems [2].

Modern virtual fencing systems consist of a solar-powered collar relying on a highly accurate GNSS tracking module to dynamically enforce virtual boundaries. The tracking module is responsible for triggering electric impulses only when an animal trespasses a proximity warning zone that is signaled by an audio-controlled tone. Therefore, the use of virtual fencing is based on the animal’s learning ability to avoid electric pulses and react to audio warnings [1,2]. This technology has demonstrated potential to modify animal trajectories and enhance grazing management [2].

The use of virtual fencing has multiple applications. The most obvious implementation is the containment of livestock movement while minimizing labor needs [1]. Other applications include targeted grazing for brush and weed control [3], managing prescribed fires [4], planning fuel breaks [5], enhancing wildlife habitat [6], and excluding livestock grazing from preferred sensitive areas [7]. More recent work evaluated the herding of cattle across rangeland pastures [8] and applications to monitor forage utilization [9]. Previous studies collectively suggest that animals can be trained to respond to a virtual fencing system without adverse effects on animal welfare [2,10,11].

Animal learning and adaptation to virtual fencing vary between individuals and training methods. Lomax et al. [12] reported a 6.5-fold variation in the number of pulses when Holstein-Friesian cows were trained to graze in restricted areas. Hamidi et al. [13] compared three approaches to evaluate animal learning and found large variations among individuals. The authors reported that the learning process can be partitioned into a first phase where Fleckvieh heifers learned to avoid electric pulses and a follow-up phase where heifers refined their ability to modify trajectories following audio warning signals. Aser et al. [14] trained Angus cows to Nofence^®^ virtual fence collars, reporting up to a 6-fold difference in audio warnings and a 1.6-fold difference in electric pulses during the training phase. Interestingly, the study reported a significant drop and much lower variation in the number of warnings and pulses among individuals in subsequent phases of the study.

Nyamurekung’e et al. [15] also reported up to a five-fold variation in the number of pulses and up to a 25-fold variation in the number of warning tones among nursing Brangus cows trained to a virtual fencing system in a relatively small corral. Furthermore, the relative number of electric pulses and ratio of pulses to audio warnings varied significantly among animals. Therefore, it is hypothesized that cows that exhibit lower ratios of electric pulses to audio warnings may learn faster and require less reinforcement to achieve prolonged containment rates. Moreover, behavioral traits, including changes in activity levels associated with grazing, walking, and resting, may also influence responses to virtual fencing, with more active individuals often displaying greater exploratory behavior, distinct temperaments, and broader grazing patterns [16].

Individual variation in learning ability, grazing behavior, and temperament is likely to influence how cattle respond to the virtual fencing system, leading to different animal containment success rates [6]. In addition, extrinsic factors, such as the season of the year, forage availability, and weather, can affect movement patterns and the effectiveness of the virtual fencing system [2]. Technical factors such as GNSS tracking accuracy, battery longevity, and signal reliability may significantly influence system performance and data integrity [9]. Importantly, short-term training trials may not fully capture long-term behavioral adaptations or residual effects on animal behavior and grazing.

The Rarámuri Criollo cattle herd managed at the Corta Madera Ranch represents a unique genetic resource [17] known for its adaptability to harsh environmental and nutritional conditions [18]. Research has shown that Rarámuri cattle exhibit wider grazing distributions, greater mobility, and reduced dependency on riparian areas compared to traditional British breeds such as Angus [19]. Their calm disposition, small frame, and heat tolerance further reduce pressure on fragile semi-arid rangelands in the Southwestern U.S. [18]. These traits make Rarámuri cattle an ideal model for evaluating virtual fencing as a behavior-based tool to guide livestock distribution in shrub-dominated landscapes.

This study hypothesizes that cows with increased movement rates due to more proactive behavioral tendencies will exhibit greater interactions at virtual fence boundaries compared to cows with less proactive behavioral tendencies. Additionally, the study further considers how changing environmental conditions, especially temporal weather patterns and forage availability, may affect grazing behavior and distribution, and the response of animals to virtual fencing. The objective of this case study is to examine the learning and behavioral responses of Rarámuri Criollo cattle to virtual fencing and evaluate likely associations with grazing patterns in a chaparral rangeland context.

## 2. Materials and Methods

The study was conducted from 16 May to 17 June 2023, at the Corta Madera ranch in Pine Valley, San Diego County, CA, USA (32.86199° N, −116.56668° W). All animal handling procedures were evaluated, approved, and carried out in accordance with the Animal Care and Use guidelines of New Mexico State University (Protocol # 1618-001).

### 2.1. Study Site and Environment

**Figure 1:**
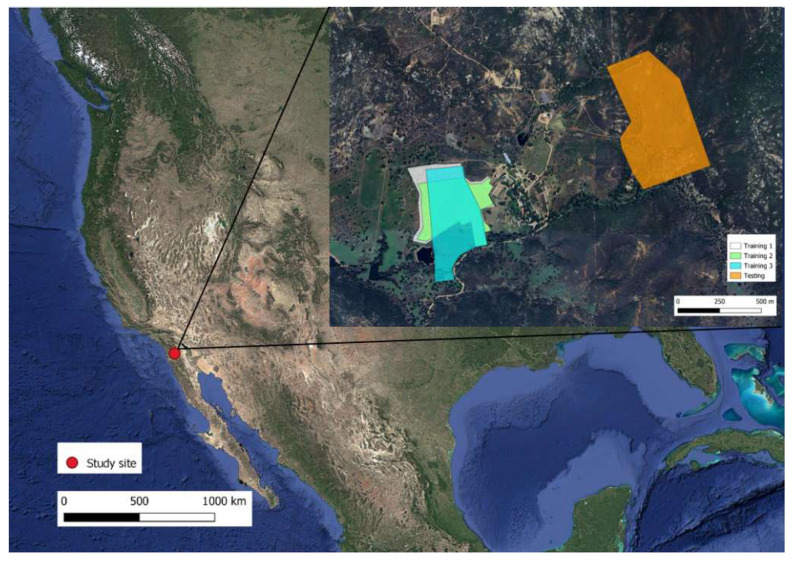
Location of the study site and paddocks used in virtual fence trials at the Corta Madera Ranch in Pine Valley, CA, United States. The map shows virtual fence enclosures used during training and testing phases, respectively.

**Figure 2:**
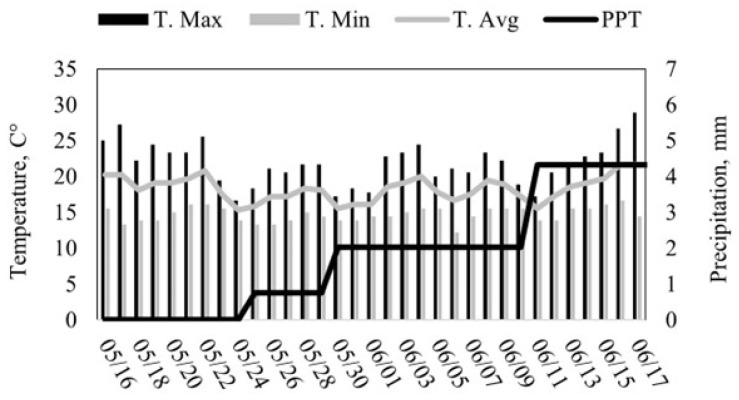
Daily temperature and precipitation during the training and testing phase. The solid vertical bars represent the maximum (Black) and minimum (Grey) temperature, while the solid lines indicate the daily average temperature (Grey) and cumulative precipitation (Black) (https://www.weather.gov, accessed on 12 October 2023).

The Corta Madera Ranch operates a cow–calf production system of Rarámuri Criollo cattle on a combination of private and US Forest Service rangeland (Figure 1). The ranch is located at an elevation of 1170 m above sea level and has a typical Mediterranean climate with hot, dry summers and mild, wet winters [20]. The historical average temperature is 17.5 °C. December is the coldest month of the year with an average temperature of 5.6 °C. August is the hottest month of the year with an average temperature of 32.5 °C. The mean annual precipitation at the site is 331 mm, mostly concentrated from December to March. During the study, the average daily temperature was 17 °C, with a minimum of 10 °C and a maximum of 30 °C (Figure 2). The average daily solar radiation (Direct Normal Irradiance) during the study was 9.3 KWh/m^2^/day, with a maximum hourly solar radiation of 23.9 of KWh/m^2^/day (https://nsrdb.nrel.gov/, accessed on 17 July 2025).

The landscape of Corta Madera is dominated by flat-topped hills. The dominant plant communities include a variety of annual and perennial grasses and forbs [20], including Mountain brome (*Bromus carinatus*), Thurbers needlegrass (*Achnatherum thurberianum*), Soft chess (*Bromus hordeaceus*), California oatgrass (*Danthonia californica*), California buckwheat (*Eriogonum fasciculatum*), desert needlegrass (*Stipa speciosa*), and foxtail fescue (*Vulpia myuros*). Upland chaparral shrubland vegetation is abundant at this site [20]. Estimates of forage production (https://rangelands.app/, accessed on 12 October 2023) for the study site in 2023 varied from 2287 to 1209 kg of dry matter per hectare. Forage analysis (NIRS, Dairy One, Ithaca, NY, USA) of composite samples (*n* = 4) of dominant dormant grasses had 4.1 ± 0.26% Crude protein, 77.5 ± 0.8% Neutral detergent fiber, 51.3 ± 1.4% Total nutrient digestibility, and 0.70 ± 0.04 Mcal kg^−1^ Net energy for lactation, respectively.

### 2.2. Study Design, Animal Response Groups, and Pastures

**Figure 3:**
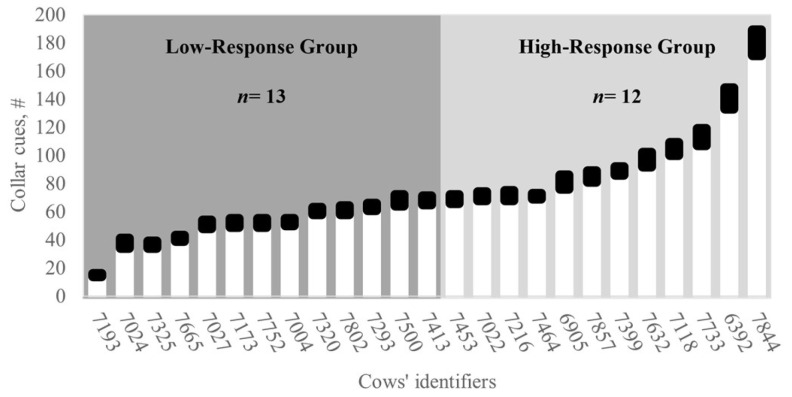
Low- and High-response groups used to assess relationships between virtual fence cues and grazing behavior variables of nursing Rarámuri Criollo cows grazing on chaparral rangeland. High-response individuals (i.e., resistant responders) and Low-response individuals (i.e., active responders) respectively had greater number (#) of collar cues and lower number (#) of collar cues compared to the median of virtual fence collar cues registered during training. The total number of collar cues included the number of audio warnings (White bars) and electric pulses (Black bars) emitted by the collars.

The study followed a completely randomized design divided into two phases: a 14-day training phase and an 18-day testing phase, respectively. Two virtual fence response groups (hereafter referred to as response groups) were predefined and compared based on the preliminary number of collar cues (audio warnings + pulses) emitted during the training and were named High- and Low-response groups, respectively. The classification considered the median number of audio warnings and pulses registered per collar (Figure 3). Cows that had fewer cues than the median were assigned to the Low-response group (i.e., active responders), while those cows with cues greater than the median were assigned to the High-response group (i.e., resistant responders).

This classification enabled us to assess behavioral and grazing patterns’ differences of cows with contrasting numbers of collar audio warnings, electric pulses, and degree of exposure to virtual fence boundaries. Pastures used for the training and testing phases are described in Table 1.

**Figure 4:**
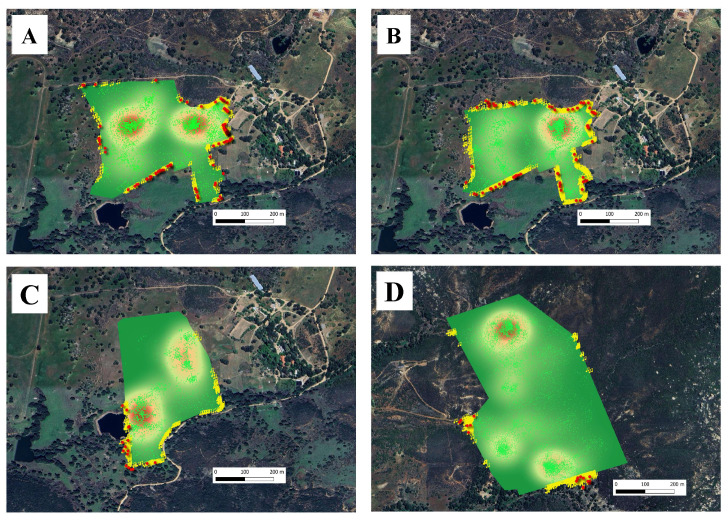
Heatmaps of cattle grazing distribution, GNSS positions (Green marks), audio warnings (Yellow marks), and pulses (Red marks) in virtual fence enclosures used during the (**A**) Training phase A, (**B**) Training phase B, (**C**) Training phase C, and (**D**) Testing phase, respectively. The characteristics of virtual fence enclosures are shown in Table 1.

The Training Phase was conducted between 16 and 29 May 2023. The phase was subdivided into three subphases to allow applying a step increase of virtual fence pressure across three enclosures (Table 1). Briefly, in Training Phase A (16 May–20 May), naïve cows were introduced to the virtual fencing system using a 166-hectare enclosure (Figure 4A). The virtual boundary was positioned 5 m inside the permanent perimeter fence, providing a gentle exclusion zone between the physical and virtual containment areas. During this phase, cattle learned to associate audio warnings with the subsequent electrical pulses from the collars while using the perimeter fence as a visual reference. In Training Phase B (21 May–25 May), a smaller enclosure was created using a 125.5-hectare virtual fence polygon. This design increased fencing pressure on the cows while minimizing reliance on the visual reference provided by the perimeter fence (Figure 4B). The configuration change in this phase also forced cattle to increasingly depend on audio warnings to avoid electric pulses, regardless of the location where warnings were received. Consequently, cattle started to alter their trajectories in the absence of the visual reference offered by the permanent fence. In Training Phase C (26 May–29 May), the virtual fence enclosure was adjusted again in terms of shape, location, and size (Figure 4C). During this final training phase, cattle further learned to rely on audio warnings to alter their trajectories and avoid electric pulses, with a configuration that differed from those in previous Training Phases A and B. The transition to the new pasture configurations used in the Training Phases A, B, and C took place on 16 May, 21 May, and 26 May, respectively.

The Testing Phase took place from 31 May to 17 June 2023, lasting 18 days. A 305-hectare enclosure (Table 1) of irregular shape was used (Figure 4D). During the testing phase, cattle were exposed to a larger and more diverse grazing environment compared to the pasture used for the training phase. This exposure allowed testing the residual effects of virtual fencing and its consequences on animal movements and behavior. The movement rate, grazing patterns, behavior, and interactions of cows with the new virtual boundaries were monitored to determine whether the learning acquired during training persisted when cows were transferred and managed more extensively on a US Forest Service allotment.

Twenty-five nursing Rarámuri Criollo cows were trained to respond to Nofence^®^ virtual fencing collars. However, five of the deployed collars experienced equipment failure during the study. The primary technical issues included collars going offline due to reduced cellular connectivity and battery controller malfunctions reported by the manufacturer that caused unexpected collar shutdowns. These collar failures resulted in incomplete datasets.

The cattle had free-choice access to graze rangeland forages available in the study paddocks, consisting of a mixture of native and naturalized grasses, forbs, and shrubs. Fresh, clean drinking water was supplied from natural ponds and permanent water troughs. Natural shade was available across study pastures. Additionally, cows had access to self-feeding low-moisture molasses and protein (CP 30%) supplements offered in tubs.

### 2.3. Virtual Fencing System

Nofence^®^ collars (https://www.nofence.no, accessed on 1 May 2023) are equipped with a micro-solar panel system and a 3.6 v, 20 Ah rechargeable Lithium battery, ensuring continuous power supply throughout the study. Each collar weighs 1.45 kg and depends on LTE Cat-M1 or 2G/4G (GSM) communication to receive and register polygon configurations (downlinks) and transmit GNSS positions and tri-axial accelerometer data (uplink). Two complementary data transmission protocols were used. A 15 min interval allows transmission of collar data in near-real time when collars have access to reliable cellular coverage. Also, a store-on-board module registers data at 30 min intervals to transmit stored data less frequently when cellular coverage is suboptimal. The GNSS tracking system integrates GPS and GLONASS satellite constellations to provide high-precision monitoring of position, heading, and speed. Each collar has a unique serial number that is used to identify the different messages transmitted by the collar. A summary of the type and number of messages transmitted by collars is shown in Table 2.

Virtual fence polygons were set using the Nofence^®^ phone App, operating either with IOS or Android systems. The polygons define three spatial and temporal management zones. The Containment Zone represents the inclusion zone where animals roam freely without receiving any collar conditioning cues or stimuli. The Cueing Zone represents the edge of the safe containment zone. In this area, audio warning tones with a rising pitch are progressively emitted (82 dB at 1 m), followed by a sequence of mild electric impulses (0.2 J at 3 kV duration = 1.0 s) only if animals do not alter their trajectories upon being warned by an audio tone. Conversely, if animals either reduce their travel speed or alter their orientation away from the virtual fence, the warning pitch slows or stops immediately. No further cues, with or without subsequent stimulus, were imposed. Up to three audio warnings followed by electric pulses can be emitted before collars shut off when animals do not respond as directed by stimuli (i.e., when the animal escaped the containment zone). The sequence and timing of audio tones and electric pulses allow the associative learning of a stimulus with a preceding cue, making the spatial and temporal presence of virtual fence boundaries predictable, avoidable, and trainable. The Nofence^®^ collar has two operating modes. In Teach mode, the animals only need to turn their heads slightly to reduce the pitch intensity, which stops the warning tone and avoids receiving an electric pulse. In the Operating mode, animals must turn away from the virtual boundary and walk approximately 2 m back into the containment zone to suppress further auditory cues. After the 20th audio cue in absence of a subsequent electric pulse, the collar automatically switches from the teach mode to the operating mode. Finally, the Excluded Zone represents the area trespassed beyond the containment zone where collars remain in the escape mode until reentering the containment zone. Collars continue to report position and activity messages, but not collar cue messages. In cases of a cueing zone breach, escape notifications are automatically reported in the user’s App.

### 2.4. Data Collection and Calculations

Data collection focused on three categories. The Collar variables included key metrics related to the interaction of animals with the virtual fence boundaries. The variables of interest included containment rate percentage, the number of pulses emitted, the number of warnings received, and the pulse-to-warning ratio. These data were used to evaluate how effectively cattle learned and progressively adapted to the virtual fencing system. The Cattle movement variables were calculated using the GRAZEACT tool [21], which is an open-access JAVA software used to calculate the area explored (ha d^−1^), sinuosity index (0 = most sinuous; 1 = straight path), and walking distance (m d^−1^) of collared animals. The GRAZEACT tool uses a minimum convex polygon (MCP) to calculate the area explored. The straightness index is used to calculate path sinuosity [22]. The walking distance is determined by using the Pythagorean Theorem to calculate segment distance between consecutive GNSS positions. Lastly, the Behavioral variables were categorized into three temporal time frames, including daily, daytime, and nighttime activity. Animal velocity determined from GNSS data was used to determine time budgets for grazing, resting, and traveling [21]. Thresholds to discriminate between resting and grazing and grazing and traveling were 2 and 8.5 m/min, respectively. We assessed how cattle allocated their daily time budgets for resting, grazing, and walking throughout the day and whether their activity patterns were affected by prior virtual fence experiences.

### 2.5. Statistical Analysis

Separate statistical analyses for training and testing trials were performed using SAS 9.4 software (SAS Institute Inc., Cary, NC, USA). Least Squares ANOVA using repeated measures mixed models [23] was used to test the independent effects of the animal response group, trial day, and the interaction between the animal response group and trial day on collar variables, animal movement, and animal behavior. Cows within each response group were treated as the subject. The covariance structure was first-order autoregressive AR (1). A Kenward-Roger adjustment was applied to the denominator degrees of freedom. The statistical model was structured as follows:

Y_ijk_ = μ + C_i_(T_j_) + T_j_ + D_k_ + T_j_D_k_ + e_ijk_ (1)

where Y_ijk_ is the dependent response variable, μ is the overall mean, C_i_ is the random effect of the *_i_* cow within the *_j_* response group, T_j_ is the fixed effect of the *_j_* response group (High or Low), D_k_ is the fixed effect of the *_k_* trial day, T_j_D_k_ is the interaction between the *_j_* response group and *_k_* trial day, and, e_ijk_ is the residual error term.

The significance alpha level of the ANOVA test was set at *p* ≤ 0.05. Post-hoc significant effects were further evaluated using LSD test (*p* ≤ 0.05).

## 3. Results

This section presents the results for collar variables, animal movement, and animal behavior detected during the training and testing phases, respectively.

### 3.1. Training Phase

#### 3.1.1. Collar Variables

There was an interaction (*p* = 0.01) between response groups and training days for the number of audio warnings (Table 3), with more audio warnings for the high- vs. low-response group (*p* < 0.0001) on a particular training day (*p* < 0.0001). Likewise, containment by the virtual fence and number of electric pulses differed (*p* < 0.006) between the response groups and among training days (Table 3). The ratio of electric pulses to audio warnings was not different (*p* = 0.40) between response groups but differed (*p* < 0.0001) among training days (Table 3).

**Figure 5:**
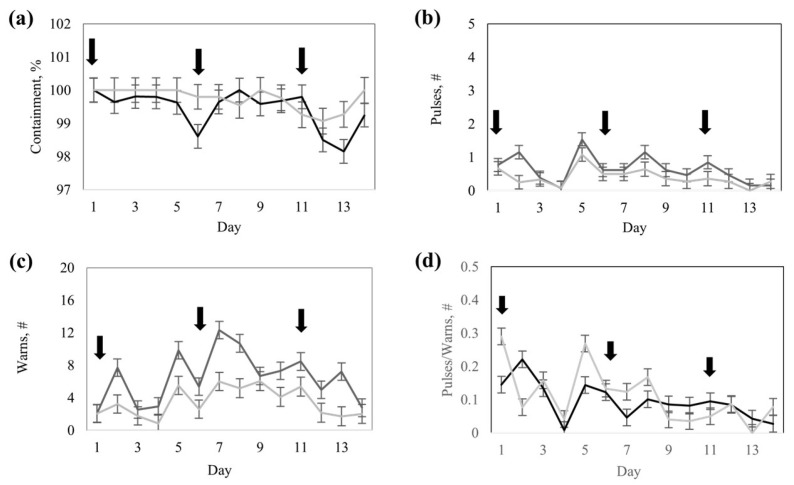
Response to virtual fencing of Rarámuri Criollo cattle classified into High- (Black) or Low- (Grey) response groups during the training phase: (**a**) Containment, (**b**) Number (#) of pulses. (**c**) Number (#) of warnings, and (**d**) Ratio of number (#) of pulses to warnings. Error bars represent standard error of the mean. Black arrows represent the days when virtual fence boundaries were modified.

The low-response group cows tended (*p* < 0.08) to exhibit greater containment than the high-response group cows (Table 3). The high-response group had a greater number of pulses than the low-response group (Table 3). The number of pulses had peaks on days 5, 8, and 11 (Figure 5). The number of audio warnings followed a similar pattern, increasing in frequency as training progressed (Figure 5). The number of warnings was greater for the high-response group compared to the low-response group (Table 3) on days 2, 5, 7, 8, and 13 (Figure 5). The ratio of pulses to warnings was greater on the first days compared to the last days of the training and had the lowest value on day 4 (Figure 5).

#### 3.1.2. Animal Movement

There was no significant (*p* > 0.57) interaction between the response group and day for daily activity, walking distance, area explored, or path sinuosity (Table 3). However, the area explored differed (*p* = 0.02) between response groups, whereas daily activity, walking distance, and path sinuosity did not differ between response groups (Table 3). All movement variables varied significantly (*p* < 0.0001) across training days (Table 3).

**Figure 6:**
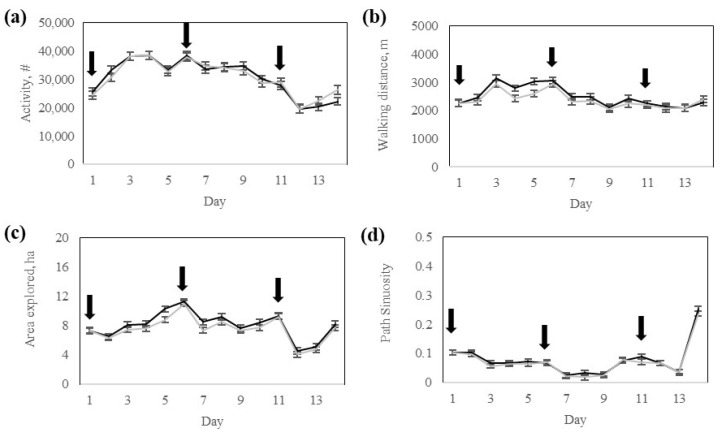
Activity of Rarámuri Criollo cattle classified into High- (Black) or Low- (Grey) response groups during the training phase: (**a**) Activity index number (#), (**b**) Walking distance, (**c**) Area explored, and (**d**) Path sinuosity. Error bars represent standard error of the mean. Black arrows represent the days when pasture boundaries were modified.

Daily activity was lower on day 1 and on the last days of the training phase (Figure 6). The walking distance was greatest on days 3 and 6 (Figure 6). The area explored was greater on days 5, 6, and 11, and lowest on days 12 and 13 (Figure 6). Path Sinuosity was lowest on days 7, 8, 9, and 13, and greatest on days 11 and 14, respectively (Figure 6).

#### 3.1.3. Animal Behavior

**Figure 7:**
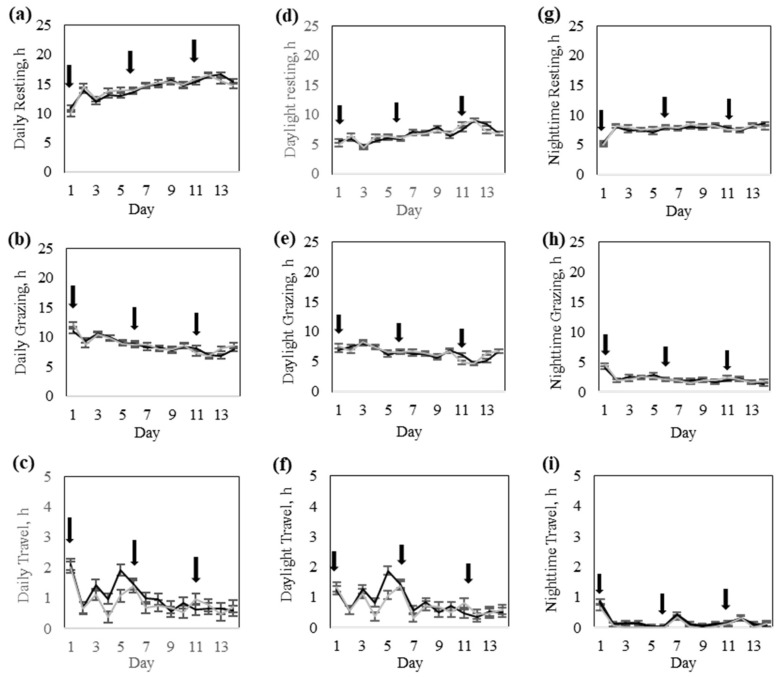
Behavior of Rarámuri Criollo cattle classified into High (Black) and Low (Grey) groups during the training phase: The daily time budget for (**a**) resting, (**b**) grazing, and (**c**) travel was partitioned into their daylight (**d**–**f**) and nighttime (**g**–**i**) components, respectively. Error bars represent the standard error of the means. Black arrows represent the days when the virtual fence pasture boundaries were modified.

There was no significant (*p* > 0.09) interaction between response groups and training day for daily time spent resting, grazing, or traveling (Table 3). Similarly, none of these behaviors differed (*p* > 0.13) between the response groups, though cows in the High-response group tended to travel more than cows in the Low-response group (Table 3). All daily behavior variables varied significantly (*p* < 0.0001) across training days (Table 3). Resting time increased, whereas grazing time decreased with increasing days of training (Figure 7). Travel time was greater on day 1 compared to all other training days (Figure 7).

There was significant interaction (*p* < 0.03) between the response group and day for daytime grazing and traveling, but not (*p* = 0.19) for resting (Table 3). All daytime behavior variables varied significantly (*p* < 0.0001) across training days (Figure 7).

Throughout the training phase, daylight resting and grazing time fluctuated between 5 and 9 h per day, with a notable shift after day 10 (Figure 7). Early in the period, grazing was the dominant behavior, but after day 11, resting increased and grazing decreased (Figure 7). Travel time during daylight hours remained consistently low, with cows spending between 0.5 to 2 h per day walking, while showing minimal temporal variation in traveling effort (Figure 7).

There was no significant (*p* > 0.26) interaction between the response group and day for nighttime resting, grazing, or traveling. Similarly, none of these behaviors differed (*p* > 0.19) between the response groups (Table 3). All nighttime behavior variables varied significantly (*p* < 0.0001) across training days (Table 3). At the start of the training period, cows exhibited a sharp increase in nighttime resting time, which stabilized around 8 h per night after day 2 (Figure 7). Nighttime grazing time showed an opposite trend, with cows grazing for approximately 4 h per night on day 1, followed thereafter by a steady decline to less than 2 h of grazing per night by the last days of training (Figure 7). Nighttime traveling time remained consistently low, with cows spending less than 1 h per night of active walking (Figure 7).

### 3.2. Testing Phase

#### 3.2.1. Collar Variables

The containment rate tended to differ (*p* = 0.09) according to an interaction between the response group and testing days. There was no significant (*p* > 0.25) interaction between the response group and days for the number of pulses, audio warnings, or the ratio of electric pulses to audio warnings (Table 4). The containment rate and audio warnings differed (*p* < 0.05) among testing days (Table 4). The number of electric pulses was not different between treatment groups (*p* = 0.87) or across testing days (*p* = 0.73). The ratio of electric pulses to audio warnings was not different (*p* = 0.51) between the response groups and did not change (*p* = 0.40) over time (Table 4).

**Figure 8:**
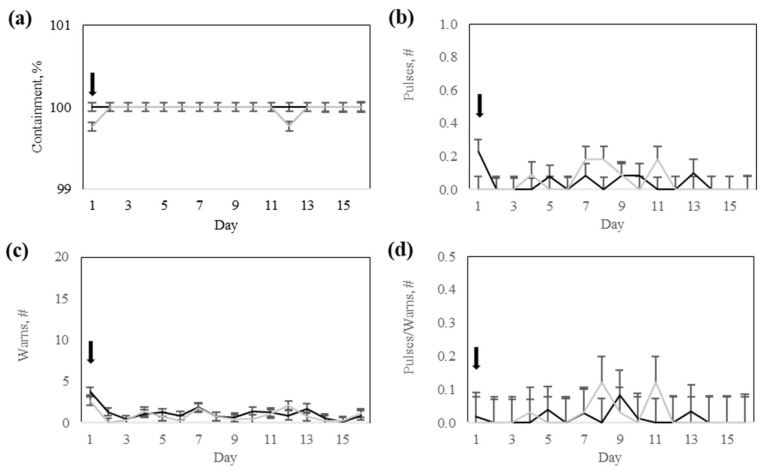
Response to virtual fencing of Rarámuri Criollo cattle classified into High- (Black) or Low- (Grey) response group during the testing phase: (**a**) containment, (**b**) number (#) of pulses, (**c**) number (#) of warnings, and (**d**) ratio of number (#) of pulses to warnings. Error bars represent the standard error of the mean. Black arrows represent the day when the virtual fence boundary was activated.

The Low-response group exhibited a decrease in containment rate on days 1 and 12, whereas the High-response group maintained a stable containment rate throughout the testing phase (Figure 8). However, both groups remained above 99.7% contained. The number of audio warnings for both groups decreased as the testing days progressed (Figure 8). The ratio of pulses to audio warnings remained consistently low for the two response groups throughout the testing phase (Figure 8).

#### 3.2.2. Animal Movement

There was no (*p* > 0.74) interaction of response group and day for daily activity, walking distance, area covered, or path sinuosity (Table 4). However, all movement variables differed (*p* < 0.0001) across testing days (Table 4).

**Figure 9:**
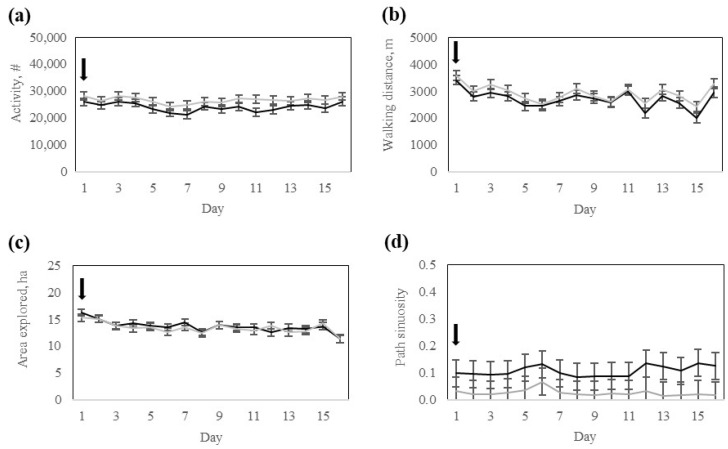
Activity of Rarámuri Criollo cattle classified into High- (Black) or Low- (Grey) response group during the testing phase: (**a**) Activity index number (#), (**b**) Walking distance, (**c**) Area explored, and (**d**) Path sinuosity. Error bars represent the standard error of the mean. Black arrows represent the day when the virtual fence boundary was activated.

The daily activity was lower on day 7 of the testing phase (Figure 9). The walking distance was greater on testing days 1 and 16 (Figure 9), whereas the area explored was greater on the first day of testing and had small peaks on days 7 and 15 (Figure 9). The daily path sinuosity differed (*p* < 0.0001) among days and remained low throughout the study (Figure 9).

#### 3.2.3. Animal Behavior

**Figure 10:**
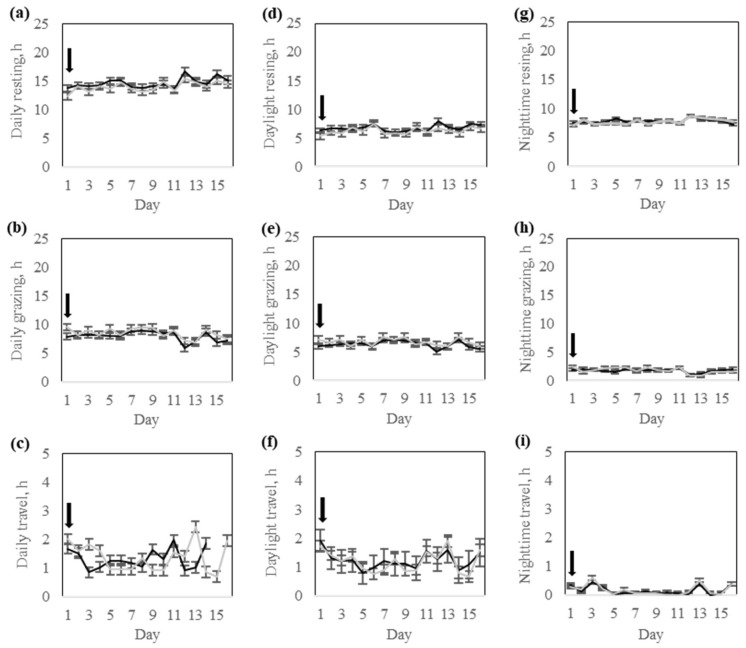
Behavior of Rarámuri Criollo cattle classified into High- (Black) or Low- (Grey) response groups during the testing phase. The daily time budget for (**a**) resting, (**b**) grazing, and (**c**) travel time was partitioned into daylight (**d**–**f**) and nighttime (**g**–**i**) components, respectively. Error bars represent the standard error of the means. Black arrows represent the day when the virtual fence boundary was activated.

There was no interaction (*p* > 0.10) between the response group and days on daily resting, grazing, or travelling time (Table 4). However, daily resting, grazing, and traveling time were different (*p* < 0.0001) across testing days (Table 4). No significant difference between the response groups (*p* > 0.12) was detected in the time spent resting and traveling (Table 4). The daily time spent grazing tended to increase (*p* = 0.08) in the Low-response group (Table 4). During the testing phase, the daily resting time remained stable at 14–17 h per day, peaking on days 12 and 15 (Figure 10). Grazing time fluctuated around 8–10 h but dropped on days 12, 13, and 15 (Figure 10). Travel time stayed below 2 h, declining to less than 1 h on days 5, 6, 7, and 12 (Figure 10).

There was no significant interaction (*p* > 0.41) between response groups and days for daylight resting, grazing, or traveling time. Daylight grazing time was greater (*p* = 0.05) for the Low-response group, whereas daylight resting time tended to be greater (*p* = 0.10) for the High-response group. Daylight traveling time did not differ (*p* = 0.61) between groups (Table 4). All daylight behavior variables varied significantly (*p* < 0.0001) across testing days (Table 4). Daylight resting time gradually increased over time, peaking on days 6, 12, and 15 (Figure 10). Daylight grazing time remained relatively stable throughout the testing phase, reaching its lowest value around day 13 (Figure 10). Daylight traveling time declined early during the first days of testing, reaching its lowest value around day 5 (Figure 10).

There was no significant interaction (*p* > 0.13) between the response group and days for nighttime resting, grazing, and traveling time. The nighttime resting, grazing, and traveling time were similar (*p* > 0.15) between the response groups (Table 4). All nighttime behavior variables varied significantly (*p* < 0.0001) across testing days (Table 4). Nighttime resting remained stable around 8–9 h per night, with a slight peak on day 12 before declining towards the end of the testing phase (Figure 10). Nighttime grazing time decreased gradually from the beginning of the testing phase, reaching its lowest value on days 12 and 13 (Figure 10). Nighttime traveling remained low throughout the testing phase, with higher values on days 3, 13, and 16, respectively (Figure 10).

## 4. Discussion

The virtual fence system was effective in keeping trained animals within the designed containment zones. Containment was successful with a rate of over 99% both during the training and testing trials. Interestingly, the level of virtual fence containment was maintained after short-term exposure to increasing virtual fence pressure, even though trained cows were nursing uncollared calves, and up to 20% of the cows had a faulty virtual fence collar. Social facilitation is known to influence both positively and negatively the responses of cows to virtual fencing during early training phases [24]. In this case study, the social facilitation of uncollared animals may have influenced the response to virtual fencing by collared animals, but not to the extent that such interference negatively affected the containment rates.

The technical challenges related to the collars also included delayed uplink reports and unsynchronized downlink configuration messages, although such delays rarely exceeded 6 h. These issues were primarily due to intermittent connectivity with GNS multicarrier networks (AT&T, Verizon, T-Mobile), a common limitation in rugged and remote areas of the U.S. However, because the virtual fencing module does not require constant GNS connectivity to function, and collars could store data onboard and transmit it every 30 min, many of the connectivity-related issues were effectively mitigated. Despite these strengths, five of the collars failed to remain operational throughout the study, resulting in incomplete datasets and the presence of unmonitored cows, which may have influenced the behavior of animals wearing functional collars. According to the manufacturing engineers, an unexpected electromagnetic interference (EMI) between components put the battery controller in a protection mode, cutting off the power supply (i.e., registering 0 volts from batteries). The issue was later resolved by introducing magnetic insulation around the battery units or replacing them with new batteries.

In this study, there was significant variation in collar responses over time and among response groups, suggesting variation in cognition, emotional reactivity, and adaptive learning strategies. During the training phase, the cows belonging to the High-response group (i.e., resistant responders) showed a clear tendency for more proactive behaviors and, therefore, received significantly more audio warnings and electric pulse stimulations than the cows in the low-response group (i.e., active responders). This response was anticipated by our study design, confirming our expectation of individual differences in movement rates and exploratory behaviors and relationships with collar cues. The observed behavioral differences were associated with more proactive behavioral tendencies, increased interactions with virtual boundaries, and a greater number of collar cues during early training for cows in the High-response group (Table 3). These results also suggest sufficient divergence in how individuals process, encode, and retrieve experiences to reinforce associations between collar warning sounds and aversive stimulations, which is the foundation for associative learning in most virtual fence applications [2]. From a behavioral perspective, this difference may relate to cows that exhibit contrasting coping styles when confronted with social or environmental challenges [16]. Less active cows tend to exhibit more passive, cautious behavior, requiring fewer reinforcements to modify actions, such as the Low-response group cows during the training phase. Conversely, more active animals may exhibit exploratory behaviors and be bolder but slower to encode negative associations between audio warnings and stimulation, thereby requiring longer exposure to virtual fencing cues. Despite the greater number of collar cues during training, the high-response group cows may encounter challenges in learning the temporal sequence and relationship between audio warnings and electric stimuli, delaying learning. A longer learning feedback may require a greater degree of reinforcement to effectively increase or reduce a particular behavior [25].

The observed behavioral patterns during training also evidence operant conditioning principles proposed by Skinner [25]. According to those principles, behaviors that are associated with a “satisfying” or rewarding outcome are likely to recur, whereas behaviors that are associated with a “discouraging” or unfavorable outcome are likely to decrease. Thus, animals associate distinguishable choices (e.g., trespassing audio boundaries) with an outcome (e.g., receiving a mild electric pulse) and use the encoded information to rectify their movements over time. However, this learning process may have differed between the Low- and High-response group cows, as discussed before. Under the conditions of this study, cows in the Low-response group appeared to have learned the virtual fence cues faster. Therefore, these cows reduced negative reinforcement from the virtual fence collars. This efficient learning may also reflect a lower sensitivity threshold to the collar cues, greater cognitive plasticity, or enhanced memory of reference [13]. Conversely, the High-response group cows may have experienced a greater degree of uncertainty, resulting in a more persistent exploration of the virtual boundary until reinforcement was consistently applied.

Additionally, cows in the High-response group significantly altered their behavior from training to the testing phases, which may partially be explained by avoidance learning. Early during the training phase (Table 3), cows in the High-response group experimented with repeated cues and electrical pulses, which may trigger a state of alertness along with anxiety-like behaviors. Such responses are known to inhibit searching and exploration as animals seek to avoid unpredictable or negative outcomes [26]. Over time, this could explain the decline in activity and increased resting behavior observed in the High-response group cows during the testing phase (Table 4). Avoidance behavior in cattle is not merely a reflection of avoiding discomfort but also a cognitive strategy to minimize uncertainty [26]. Animals in unpredictable conditions may prioritize safety by reducing exposure to unfamiliar or potentially threatening situations, even if the risk is no longer present [27]. In the context of virtual fencing, this response could result in greater spatial restriction or reduced willingness to explore new areas near virtual fence boundaries. This cautious approach may influence grazing distribution, as it may result in underutilization of certain pasture areas if animals associate those regions with risk of negative pulse stimulations.

Social learning may also play a role in adaptation to the virtual fence [24], as discussed earlier. In group-managed cattle, observational learning is the mechanism through which individuals learn to interpret given cues by observing peers. It is plausible that cows in the Low-response group benefited from the early exposure to herd mates in the High-response group, which had a greater degree of exposure to the virtual fence during training. Thus, a few leading individuals in a cattle herd may stimulate similar responses in other cautious individuals, promoting herd cohesion and faster adoption of learned behaviors [28].

The group-by-day interaction observed for audio warnings further supports the notion that the cows in the High-response group had a slower but steady learning trajectory, eventually achieving similar containment performance over time. These dynamics are critical when implementing behavior-based systems like virtual fencing in commercial settings, as they highlight the need for flexible adaptation periods and differential training protocols based on individual responsiveness. Precision livestock management strategies can leverage such behavioral profiles to predict training pressure needs, reduce overstimulation, and improve animal welfare. One practical approach to incorporating adaptive training protocols, particularly in large commercial herds, is to monitor responses and extend the duration of the training phase based on a defined benchmark outcome. This allows animals that require more time and reinforcement to be trained to the same success level as more responsive or rapidly adapting individuals. Rather than exposing all animals to a fixed number of training days, training efficiency could be improved by developing a monitoring routine based on the ratio of electric pulse responses to audio warning tones. This data-driven approach would allow for individualized training progression, ensuring more consistent learning outcomes across the herd.

Interestingly, despite differences in electric pulse stimulus exposure during the training phase (Table 3), both groups exhibited comparable activity, walking distance, and time spent grazing, resting, or traveling during the testing phase (Table 4). This finding indicates that the core structure of daily activity was not disrupted by prior fencing experiences, even in animals that received more stimulation during early transition phases. However, the High-response group cows explored a greater area during the training, potentially reflecting greater exploration behavior triggered by their initial exposure to the virtual fence. From a behavioral ecology perspective, this response may represent a greater motivation to search for alternative resources or to find alternative routes in response to increased containment pressure, particularly in a novel environment. Upon the progression of training, behaviors were likely stabilized across the two response groups. Consequently, no response group-by-day interactions or group differences were found during the testing phase, suggesting that once the virtual fence is properly learned, both response groups equally maintained a high containment rate with a minimal number of audio warnings or pulse reinforcements. This behavioral consistency indicates that regardless of the initial strategy for learning, all cows effectively learned to respond to the virtual fence cues while adapting their movement patterns and grazing accordingly. Subtle behavioral differences persisted as the Low-response group cows exhibited higher daily activity and walking distance, while the High-response group cows spent more time resting and slightly less time grazing during the testing. These long-term effects may stem from previous experiences with the virtual fence, as discussed earlier.

The behavioral phenotypes observed during testing may also reflect intrinsic foraging styles of Rarámuri cattle, as documented in previous research at this site [19]. Some cows consistently exhibit wider distribution range and more exploratory behavior, while others may have prioritized resource efficiency, safety habitats, and reduced movement. These consistent individual differences among individuals may explain the occurrence of behavioral syndromes in cattle [16] and may interact with virtual fencing by influencing how animals respond to the perceived risks and space restrictions imposed by the auditory fence. In future implementations, integrating temperament assessments or behavioral profiling could help identify individuals more likely to need a different training exposure or additional support.

The genetic history and behavioral traits of Rarámuri Criollo cattle may have also influenced the success of virtual fencing in this study [17]. The Rarámuri Criollo cattle is a calm animal known to display adaptive behaviors well-suited to rugged terrain, hot temperatures, and patchy environments, such as the chaparral pastures at Corta Madera ranch [19]. Their tendency for broad spatial distribution and opportunistic foraging behavior likely contributed to the high containment rates and behavioral plasticity seen in previous studies [18]. The adaptive capacity of Rarámuri Criollo cattle may be especially valuable when introducing cattle to new management technologies such as virtual fencing.

Daylight and nighttime comparisons also revealed that most behavioral variation among individuals occurred during daylight hours. This is expected due to the circadian and crepuscular nature of grazing and resting, respectively. Grazing time usually concentrates during cooler morning and evening hours, while resting dominates during nighttime hours [29]. However, diurnal grazing patterns may also shift with increasing daylight temperatures in summer, explaining the extension of grazing by Rarámuri Criollo cows during nighttime hours. Barto et al. [30] also documented increased nighttime grazing of Corriente criollo cattle during the summer in higher elevation pastures in Arizona. Furthermore, the nighttime resting behavior remained stable between groups, suggesting that prior differences in virtual fence use did not disrupt resting rhythms or cause prolonged stress or hypervigilance. This finding reinforces previous work suggesting that once the virtual fence is learned, dynamic changes in boundaries are highly respected without bringing additional cognitive or emotional burden or adverse effects on animal welfare [11].

Forage chemistry dynamics may also help explain some of the behavioral outcomes observed. During trials, the forage quality of dormant annual grasses likely declined with the advancement of plant maturity. Likewise, low crude protein, digestibility, and energy content (see Section 2) may have increased the motivation to graze selectively. In this study, foraging paths were not strictly linear, suggesting that some degree of path tortuosity was due to animal selectivity. This finding also supports the theory that Rarámuri Criollo cows show behavioral plasticity when exposed to nutritionally challenging conditions, as in this study.

Moreover, the decline in forage quality may influence how cows perceive the value of exploring the landscape. For cows in the High-response group, previous exposure to the virtual fencing stimuli may have discouraged their search movement even in response to low forage quality. On the other hand, the Low-response group cows may have perceived greater benefit in seeking better resources across the containment area, which may explain their greater movement rate during the testing phase. Better understanding of likely interactions between forage abundance and quality, spatial memory, virtual fence reinforcement, and behavioral tendencies requires additional behavioral and nutritional research.

## 5. Implications

This study provides further insight into training cattle to use virtual fencing extensively and applying the technology to manage grazing systems without disrupting typical behavioral patterns. The success of the Nofence^®^ collars in maintaining a high containment rate while allowing cattle to express their typical time budgets for grazing, resting, and traveling aligns with the goal of applying low-stress precision-based livestock management strategies on rangelands. Additionally, a better understanding of the basis for likely differences in the learning response to virtual fencing could inform behavior-based protocols to enhance animal training and technology adoption rates. Future adoption may also come through improved virtual fencing systems capable of operating under limited coverage and reliable power sources. Interest in virtual fencing in arid and semi-arid rangelands such as those of the Southwestern United States is driven by the desire to reduce expensive fencing infrastructure, which is aligned with emerging rangeland management sustainability goals. Most rangelands are characterized by difficult terrain, increased woody encroachment, limited water resources, and uneven forage distribution. Permanent fencing is costly, inflexible, and often ecologically disruptive. In contrast, virtual fencing offers a more dynamic approach to control grazing pressure of heterogeneous resources [2], with potential practical applications to exclude grazing from sensitive areas or concentrate livestock grazing on targeted areas [4,5].

Despite the demonstrated applications, future adoption at scale will require attention specific to the selected virtual fence operating systems, the users’ operation, animal training protocols, and integrations with other technologies. For example, GNSS combined with accelerometer data could be used to improve virtual fence monitoring systems. The integration of machine learning into livestock monitoring has already begun showing promise in differentiating among multiple behaviors of free-range cattle from Nofence^®^ collars [31]. This improvement may offer new opportunities to proactively apply behavior-based management systems to enhance virtual fence applications [2]. Lastly, while this study focused on behavioral responses, future work should integrate physiological stress indicators (e.g., cortisol concentrations, heart rate variability) to fully assess animal well-being in response to distinct virtual fence applications. Monitoring behavioral data along with physiological parameters would strengthen the foundation for welfare-based guidelines, especially in systems emphasizing autonomy and self-regulated movements through virtual fencing. The integration of temperament assessments or behavioral profiling could also help identify individuals more likely to need a different training exposure or additional support.

## 6. Conclusions

This case study supported the hypothesis that differential learning responses to virtual fencing are shaped by distinct behavioral tendencies of cattle. More active individuals typically had greater early interaction with the virtual fence. The Rarámuri Criollo cattle effectively adapted to virtual fencing, achieving a high containment rate. The study highlights the flexibility of the technology for application in extensive beef production systems in the southwestern United States.

## Figures and Tables

**Table 1:** Characteristics of virtual fence enclosures.

ID	Study Phase	Start Date	End Date	Area ha	SD ^a^ ha cow^−1^	VFL ^b^ m	VFD ^c^ m cow^−1^	VFP ^d^ m/ha cow^−1^
1	Training A	16 May 2023	20 May 2023	166.0	6.6	2247	89.9	13.5
2	Training B	21 May 2023	25 May 2023	125.5	5.0	1938	77.5	15.4
3	Training C	26 May 2023	29 May 2023	145.4	5.8	1698	67.9	11.7
4	Testing D	31 May 2023	17 June 2023	305.0	12.2	2319	92.8	7.6

^a^ SD = Stocking density; ^b^ VFL = Virtual fence length; ^c^ VFD = Virtual fence density; ^d^ VFP = Virtual fence pressure.

**Table 2:** Type and number of messages transmitted by the Nofence^®^ collars.

Type	Frequency	Description	Observations
Poll	15 min	Position data	68,973
Seq	30 min	Position data	37,379
Seq2	30 min	Environmental	37,389
Client_Warning	After an audio warning	Positional data	2246
Client_Zap	After an electric pulse	Positional data	204

**Table 3:** Effects of virtual fencing on collar variables, movement, and behavior of Rarámuri Criollo cows during training.

	Group ^1^			*p*-Value		
	High	Low	SE	Group	Day	G × D
Collar variables ^2^						
Containment, %	99.44	99.75	0.10	0.08	0.0055	0.56
Pulse, #	0.60	0.37	0.05	<0.0001	<0.0001	0.58
Warnings, #	6.17	3.36	0.37	<0.0001	<0.0001	0.01
Pulse/Warnings	0.09	0.10	0.01	0.40	<0.0001	0.37
Movement variables						
Daily Activity Index	30,664	30,715	791	0.96	<0.0001	0.57
Walking distance, m	2503	2377	61	0.14	<0.0001	0.59
Area Explored, ha	8.04	7.50	0.16	0.02	<0.0001	0.76
Path Sinuosity	0.08	0.07	0.00	0.10	<0.0001	0.96
Daily behavior variables						
Resting, h	14.32	14.39	0.16	0.76	<0.0001	0.30
Grazing, h	8.64	8.73	0.13	0.62	<0.0001	0.34
Travel, h	1.03	0.87	0.07	0.13	<0.0001	0.09
Daylight behavior variables						
Resting, h	6.70	6.68	0.10	0.87	<0.0001	0.19
Grazing, h	6.46	6.56	0.09	0.43	<0.0001	0.03
Travel, h	0.84	0.75	0.05	0.16	<0.0001	0.02
Nighttime behavior variables						
Resting, h	7.62	7.73	0.12	0.51	<0.0001	0.26
Grazing, h	2.19	2.17	0.09	0.86	<0.0001	0.32
Travel, h	0.20	0.12	0.04	0.19	<0.0001	0.86

^1^ Animal response group; ^2^ #: number recorded. The *p*-values indicate significance for the effect of animal response group (Group), day (Day), and their interaction (G × D).

**Table 4:** Effects of virtual fencing on collar variables, movement, and behavior of Rarámuri Criollo cows during testing.

		Group ^1^			*p*-Value		
		High	Low	SE	Group	Day	G × D
Collar variables ^2^							
	Containment, %	100.00	99.97	0.05	0.15	0.05	0.09
	Pulse, #	0.04	0.05	0.02	0.87	0.73	0.52
	Warnings, #	1.17	0.90	0.17	0.27	<0.0001	0.63
	Pulse/Warnings	0.01	0.02	0.01	0.51	0.40	0.25
Movement variables							
	Daily Activity Index	24,037	26,649	1009	0.07	<0.0001	0.74
	Walking distance, m	2701	2914	117	0.20	<0.0001	0.82
	Area Explored, ha	13.12	13.40	0.39	0.61	<0.0001	0.97
	Path Sinuosity	0.11	0.03	0.05	0.29	<0.0001	0.31
Daily behavior variables							
	Resting, h	14.66	14.06	0.27	0.12	<0.0001	0.42
	Grazing, h	8.03	8.57	0.21	0.08	<0.0001	0.45
	Travel, h	1.39	1.37	0.06	0.85	<0.0001	0.84
Daylight behavior variables							
	Resting, h	6.71	6.28	0.19	0.10	<0.0001	0.40
	Grazing, h	6.34	6.76	0.15	0.05	<0.0001	0.41
	Travel, h	1.24	1.21	0.05	0.61	<0.0001	0.75
Nighttime behavior variables							
	Resting, h	7.83	7.78	0.08	0.64	<0.0001	0.16
	Grazing, h	1.78	1.81	0.08	0.81	<0.0001	0.13
	Travel, h	0.15	0.17	0.02	0.60	<0.0001	0.72

^1^ Animal response group; ^2^ #: number recorded. The *p*-values indicate significance for the effect of animal response group (Group), day (Day), and their interaction (G × D).

## Data Availability

Dataset available on request from the authors.
